# Functional Assays in the Diagnosis of Heparin-Induced Thrombocytopenia: A Review

**DOI:** 10.3390/molecules22040617

**Published:** 2017-04-11

**Authors:** Valentine Minet, Jean-Michel Dogné, François Mullier

**Affiliations:** 1 Department of Pharmacy, Namur Thrombosis and Hemostasis Center (NTHC), Namur Research Institute for LIfe Sciences (NARILIS), University of Namur, Namur 5000, Belgium; 2 CHU UCL Namur, Namur Thrombosis and Hemostasis Center (NTHC), Hematology Laboratory, Université catholique de Louvain, Yvoir 5530, Belgium

**Keywords:** heparin-induced thrombocytopenia, diagnosis, functional assay, platelets

## Abstract

A rapid and accurate diagnosis in patients with suspected heparin-induced thrombocytopenia (HIT) is essential for patient management but remains challenging. Current HIT diagnosis ideally relies on a combination of clinical information, immunoassay and functional assay results. Platelet activation assays or functional assays detect HIT antibodies that are more clinically significant. Several functional assays have been developed and evaluated in the literature. They differ in the activation endpoint studied; the technique or technology used; the platelet donor selection; the platelet suspension (washed platelets, platelet rich plasma or whole blood); the patient sample (serum or plasma); and the heparin used (type and concentrations). Inconsistencies in controls performed and associated results interpretation are common. Thresholds and performances are determined differently among papers. Functional assays suffer from interlaboratory variability. This lack of standardization limits the evaluation and the accessibility of functional assays in laboratories. In the present article, we review all the current activation endpoints, techniques and methodologies of functional assays developed for HIT diagnosis.

## 1. Introduction

Accurate and rapid diagnosis of heparin-induced thrombocytopenia (HIT) is essential to improve clinical management of patients. Because thrombocytopenia is rather frequent in hospitalized patients receiving heparin, clinicians must distinguish the uncommon patient with HIT among the many without [1]. Accurate diagnosis is crucial as overdiagnosis may expose the patient to alternative anticoagulant treatment conferring a significant risk for major bleeding complications. Moreover, physicians are very reluctant to reintroduce heparin in such patients [2,3]. Misdiagnosis will delay the initiation of the alternative treatment, increasing thrombotic risk and mortality [4]. Clinical scoring systems, such as the 4Ts score or the HEP score, are helpful in estimating the probability of HIT [5,6]. A low probability 4Ts score appears to be a robust means of excluding HIT [7,8] but does not rule out HIT in all cases [9,10] and may be difficult to apply [11]. Patients with intermediate and high probability scores require further evaluation [7]. HIT is often difficult to exclude or to confirm based on clinical information alone [1]. HIT diagnosis requires laboratory testing. Two types of assays are available: immunoassays and functional assays [1]. Immunoassays detect binding of anti-PF4/heparin antibodies (Ab). Functional assays or platelet activation assays investigate if these antibodies are able to activate platelets in the presence of heparin. Depending on the clinical setting, only 10%–50% of patients with positive immunoassays have platelet-activating antibodies [12]. HIT cannot be confirmed by immunoassays alone because of low positive predictive value [13]. A recent systematic review and meta-analysis concluded that only five immunoassays evaluated have a high sensitivity (>95%) and a high specificity (>90%) [14]. Moreover, optical density (OD) values of immunoassays vary among laboratories and need standardization of the OD ranges [15]. Performing a functional assay in the case of positive immunoassay is highly needed to reduce overdiagnosis and subsequent mistreatment of patients without HIT [16]. An integrated diagnostic approach combining clinical information with immunoassays and functional assays is recommended and provides a guide to decision making facing a patient suspected of HIT [5,14,17]. Making a timely and accurate diagnosis of HIT remains an important challenge because of limitations of current diagnostic tests. Functional assays considered as gold standards, i.e., ^14^C-serotonin release assay (^14^C-SRA) and heparin-induced platelet activation (HIPA)), require a highly specialized laboratory and are not widely available [18]. Several platelet activation assays avoiding limitations of previous assays have been developed. We provide an overview of all current laboratory endpoints, techniques, assay variations and results interpretation in functional assays for HIT diagnosis.

## 2. Principles of Functional Assays

**Figure 1:**
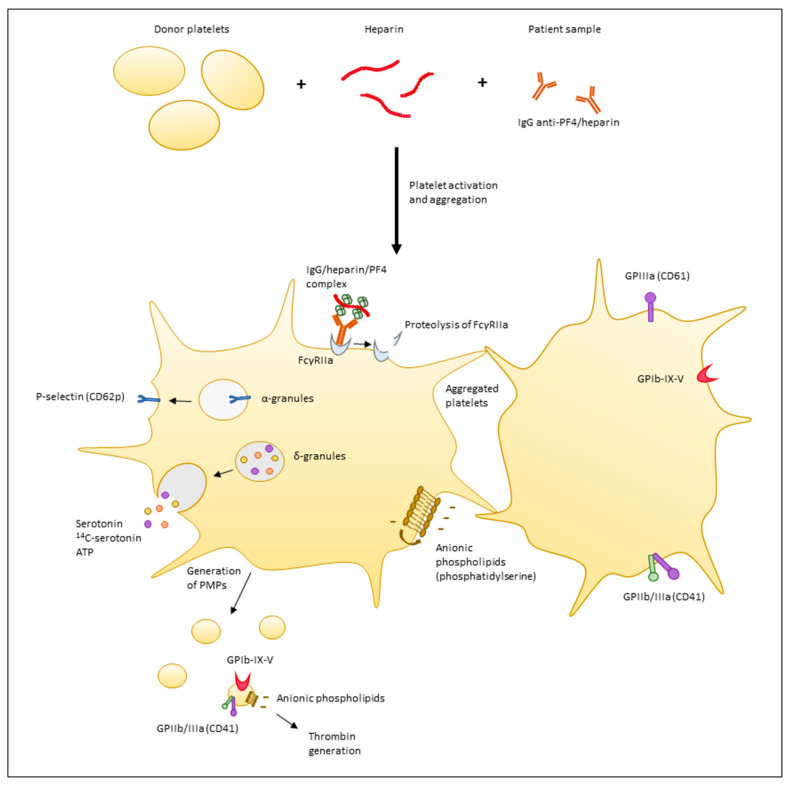
Platelet changes induced by heparin-induced thrombocytopenia (HIT) antibodies and detected in HIT functional assays. Donor platelets, heparin and the patient sample are incubated in vitro. Clinically significant antibodies lead to the formation of an antibody/heparin/PF4 complex that binds to FcγRIIa receptors on the platelet and induces donor platelets activation and aggregation. The following platelet changes are induced and may be used as endpoints in functional assays: proteolysis of the FcγRIIa receptor; translocation of p-selectin (CD62p) from α-granules to the platelet surface; release of δ-granules (dense granules) content containing serotonin; ATP and preincubated radiolabeled serotonin; generation of PMPs; procoagulant activity of PMPs with thrombin generation; translocation of anionic phospholipids such as phosphatidylserine to the outer surface by a flip-flop mechanism; and aggregation of platelets. GPIIIa (CD61) and GPIIb/IIIa (CD41) are two platelet and PMP surface glycoproteins (GP) expressed on normal platelets. GPIb–IX–V is a subunit of the von Willebrand factor receptor complex expressed on the surface of platelets and PMPs. (IgG: immunoglobulin G, PF4: platelet factor 4, ATP: adenosine triphosphate, PMPs: platelet microparticles.)

In functional tests, donor platelets are incubated with patient serum/plasma and heparin. If clinically significant patient′s HIT antibodies are present with an optimal heparin–PF4 stoichiometric ratio, this leads to the formation of a heparin–antibody–PF4 complex that will bind to FcγRIIa receptors on the platelet and induce donor platelets activation. This in vitro reaction results in platelet changes including release of α-granules and dense bodies, generation of platelet microparticles (PMPs), upregulation of various membrane glycoproteins (GP) and ultimately platelet aggregation. These platelet changes may be used as endpoints in functional assays (Figure 1) [19].

## 3. Activation Endpoints

### 3.1. Release of Dense Granules Content

#### 3.1.1. Release of Serotonin

Almost all circulating serotonin is accumulated and stored in the dense granules of platelets [20,21]. When platelets are activated by HIT antibodies, they release serotonin in the supernatant. The measurement of the serotonin release can be used to detect HIT antibody-induced platelet activation within patient serum or plasma [22]. The first assay proposed to measure this release of serotonin as a platelet activation point in the diagnosis of HIT was the ^14^C-serotonin release assay (^14^C-SRA) [23]. To perform this assay, donor platelet-rich plasma (PRP) is pre-incubated with radioactive ^14^C-serotonin. Radiolabeled serotonin enters dense granules of platelets. After a washing procedure, washed platelets are incubated with patient serum/plasma and heparin followed by a centrifugation step where supernatants are collected and radioactivity is measured using a β-counter [22].

Alternatives to study platelet serotonin release have been described in the literature. These techniques avoid the use of a radioactive agent and measure the release of intraplatelet endogenous serotonin. The first technique is the enzyme linked immunosorbent assay (ELISA) [24]. During the incubation step, endogenous serotonin is released from dense granules to the supernatant. After a centrifugation step, the serotonin contained in the supernatant is quantified with an ELISA [24,25,26]. The second technique to quantify serotonin released in the supernatant from platelets is the high-pressure liquid chromatography (HPLC) [25,26,27] coupled to a fluorescent detector [27] or to an electrochemical detector [25]. The third technology analyses intraplatelet serotonin content using flow cytometry [28]. After the incubation step, platelets were identified using an antiCD41a monoclonal antibody that recognizes a calcium-dependent complex of GPIIb/IIIa expressed on normal platelets. Intraplatelet serotonin content was detected with an antiserotonin antibody labelling after fixation and permeabilization of platelets. Platelets activation was independently shown by annexin V binding [28].

#### 3.1.2. Release of Adenosine Triphosphate (ATP)

The platelet dense granules contain large stores of ATP [29]. ATP release from platelets during platelet activation induced by HIT antibodies can be measured using a lumiaggregometer [29]. Luciferase–luciferin reagent is added to the platelet samples, it reacts with released ATP to generate adenyl–luciferon. A chemiluminescent reaction occurs when adenyl–luciferin oxides. The light emitted is proportional to the quantity of ATP present in the aggregometer cuvette [19]. Another group reported the use of a standard scintillation counter to quantify the released ATP [30].

#### 3.1.3. Platelet Aggregation

The platelet aggregation endpoint can be measured in three different ways.

##### Visual Assessment

HIPA test is based on visual assessment of platelet aggregation in U-bottomed polystyrene microtiter wells with rotating steel balls used to agitate the platelets [31,32,33,34,35]. Donor platelets, patient serum and heparin are stirred using a magnetic stirrer. The wells are examined visually against an indirect light source at 5-min intervals. A change in appearance of the reaction mixture from turbidity (nonaggregated platelets) to transparency (aggregated platelets) is considered a positive result [36].

##### Optical Aggregometry

The heparin-induced platelet aggregation test (PAT) evaluates platelet aggregation using conventional light transmission aggregometry (LTA) [33,37]. The test principle is similar to HIPA, except that the platelet aggregation endpoint is not evaluated visually but with the use of an optical aggregometer. The reaction mixture is stirred in a cuvette at 37 °C between a light source and a photocell and the light transmission is recorded over time. Aggregation is detected by an increase in light transmission through the platelet suspension [38].

##### Impedance Aggregometry

Platelet aggregation can be detected with the heparin-induced multiple electrode aggregometry (HIMEA) [39,40,41,42,43,44]. The reaction mixture is incubated and stirred in a cuvette containing two pairs of sensor electrodes. When platelet aggregation occurs, platelets stick on the electrodes inducing an enhancement of the electrical resistance between them. This change in impedance is recorded over time in the whole blood impedance analyzer (Multiplate^®^ analyzer, Dynabyte Medical, Munich, Germany).

#### 3.1.4. Expression of Platelet Membrane Glycoproteins

During platelet activation, changes are induced in the platelet membranes with expression of surface markers. These platelet activation markers can be studied in flow cytometry with the use of fluorescent-labeled ligand [38]. Expression of anionic phospholipids and P-selectin have been proposed in the literature as identification markers of platelet activation [45,46,47,48,49,50]. During platelet activation, a membrane flip-flop mechanism translocates anionic phospholipids such as phosphatidylserine to the outer surface. Annexin V is a protein that binds with high affinity and specificity to anionic phospholipids expressed on the surface of activated platelets [19]. P-selectin is a protein stored in the membranes of the α-granules of resting platelets. Upon platelet activation, P-selectin (CD62P) is translocated to the platelet surface [19,38] and can be measured using a labelled anti-P-selectin antibody. Platelet marker antibodies are also used to identify the platelet population. Anti-CD61 and anti-CD41 recognize two platelet surface GP: platelet GPIIIa (CD61) [50] and platelet complex GPIIb/IIIa (CD41), respectively [45,46,48,51]. After the in vitro platelet activation by HIT antibodies, the platelet mixture is incubated with two fluorescent-labelled ligands: one to identify platelets and the other to detect activated platelets. The proportion of activated platelets is obtained by two-color flow cytometry.

#### 3.1.5. Generation of Platelets Microparticles

Platelets activated by HIT sera generate platelet-derived microparticles [52,53,54]. PMPs are quantified using flow cytometry. After the incubation step, platelet mixture is incubated with a fluorescent-labelled antibody to identify platelets and PMPs such as anti-CD41 [51,55,56,57] or anti-GPIbα [58], the latter monoclonal antibody links a subunit of the von Willebrand factor receptor complex (GPIb–IX–V) expressed on the surface of platelets and PMPs [19]. Annexin-V may be added to bind anionic phospholipids as a platelet activation marker [51,55,56,57]. PMPs are distinguished from platelets by their size and scatter parameters.

#### 3.1.6. Procoagulant Activity

Platelet-derived microparticles generated by HIT antibodies are procoagulant and lead to thrombin generation [53]. The procoagulant activity of HIT IgG antibodies can be measured with the thrombin generation assay (TGA) using a fluorometer [59,60]. At the end of the incubation with HIT antibodies, recombinant human tissue factor is added and coagulation is triggered with the addition of a fluorogenic substrate and calcium chloride. The peptidic fluorogenic substrate is hydrolysed by thrombin and releases a product which emits fluorescence. The fluorescence intensity is recorded over time and converted into a peak of active thrombin concentration against time [61].

#### 3.1.7. FcγRIIa Proteolysis

FcγRIIa agonists lead to cleavage of the receptor and the retention of a 32-kDa membrane bound component [62,63]. Identification of the proteolytic fragment of FcγRIIa with a Western blot analysis could serve as a surrogate marker for HIT [64]. After the incubation time, platelet activation is stopped with EDTA in the presence of protease inhibitors. Platelets are centrifuged and solubilized in lysis buffer. To measure proteolysis, lysates are separated by SDS-PAGE and detected by Western blot analysis using goat anti-human FcγRIIa with avidin–horseradish peroxidase and chemiluminescence [64]. The percentage proteolysis is determined using scanning densitometry.

#### 3.1.8. Intracellular Luciferase Cell Activity

Cuker and collaborators recently developed a new functional assay to identify cell-activating anti-PF4/heparin antibodies without need for donor platelets but using a cell-line that can be stored at −80 °C [65]. DT40 chicken B lymphocyte cells were transiently transfected to express human FcγRIIA and a luciferase reporter. The PF4/heparin/plasma mixtures are added to the resultant transgenic B-cell line. HIT immune complexes bind to B cells and cross-link the FcγRIIA which induces an intracellular signaling cascade, ultimately leading to luciferase activation, resulting in a luminescence signal. Luciferase activity is measured with a luminometer using Luciferase Assay Reagent. Data are reported as the signal induced by patient plasma relative to the absence of plasma (fold-basal) [65].

## 4. Platelets

### 4.1. Whole Blood, PRP or Washed Platelets

Donor platelets can be used in three different ways: either as washed platelets, as PRP or as whole blood.

Whole blood does not require platelet preparation after the blood drawn and is therefore more rapid to obtain than PRP or washed platelets.

To obtain PRP, a simple centrifugation of whole blood at a low speed (150 to 180 g for 10 to 15 min at room temperature) is needed [32]. PRP is sometimes adjusted to 250,000–300,000 platelets/µL [29,42,48,66]. PRP is technically less demanding than washed platelets and can be performed in a non-specialist clinical laboratory [19].

To prepare washed platelets, blood is collected into acid–citrate–dextrose (ACD) solution. ACD reduces the pH in order to prevent platelet aggregation that would occur during platelet pelleting [36]. ACD also chelates the calcium in blood preventing coagulation. A low speed centrifugation is performed to obtain PRP. Platelets are isolated from PRP by successive centrifugation steps and re-suspended in calcium- and magnesium-free Tyrode′s buffer at pH 6.3 with glucose and apyrase. The absence of calcium and magnesium allows the activation of coagulation factors and platelets to be avoided [36]. Apyrase is an enzyme that prevents adenosine diphosphate (ADP) accumulation from the platelets by degrading adenine nucleotides. It maintains platelet sensitivity to subsequent ADP stimulation that occurs during the second phase of HIT antibody-induced platelet activation [32,67]. Platelets are resuspended into calcium- and magnesium-containing Tyrode′s buffer at physiological pH (pH 7.4) without apyrase or hirudin to allow HIT-induced platelet activation [36].

Platelet washing is a time-consuming procedure requiring experience and care to avoid excessive platelet activation [19,51]. Although, the mild background platelet activation generated during the washing procedure can be advantageous in HIT assays, excessive platelet activation can occur in inexperienced hands and lead to erroneous results [19]. Washed platelets are best suited for specialist or referral laboratories assessing many HIT sera/plasma as this facilitates acquisition of sufficient technical experience to perform the assay successfully on a consistent basis [36]. In the nonspecialist clinical laboratories, the technicians may not have the experience or training to perform platelet washing properly and a PRP-based assay may be more appropriate [19].

Washed platelet-based assays are considered more sensitive and possibly more specific than PRP or whole blood-based assay tests for some biological reasons [19,36,68]: (i) the wash step eliminates possible interfering substances potentially causing heparin independent aggregation such as IgG or acute-phase proteins (e.g., fibrinogen) [69]; (ii) the high centrifugation during washing may induce platelet granule release of PF4 with greater formation of PF4/heparin antigen complexes [19]; (iii) the use of apyrase prevents platelets from becoming refractory to subsequent ADP-mediated potentiation of HIT-antibody-induced activation; (iv) the physiological calcium concentrations induce optimal IgG-mediated platelet activation [36,69].

Previous studies have compared PAT, a PRP aggregation test to SRA, a washed platelets activation assay. They suggested that PAT has lower sensitivity than SRA and may miss cases of true HIT [33,35,70,71]. In contrast, another study reported a similarly high sensitivity for PAT in comparison to SRA but with a lower specificity for PAT [72]. It is believed that when performed under controlled conditions with highly responsive platelets and with a two-point system, the sensitivity and specificity of PRP-based assay can approach or be similar to washed platelet-based assays [19,33]. More recent studies compared the performances of SRA to newer PRP or whole-blood-based tests and showed close or similar sensitivity and specificity [27,42,44,56,73]. No direct comparison of functional assays using the same patient samples with the same platelet donors using PRP and washed platelets has been described in the literature.

### 4.2. Platelet Donor Selection

It was reported that serum/plasma of a patient with HIT highly activates autologous platelets [74,75]. It has been potentially explained by a persisting high expression of FcγRIIa on platelets [75], baseline platelet activation and higher availability of PF4 in patients with acute HIT [76]. However, use of autologous PRP can be limited by the patient thrombocytopenia, hence the use of donor platelets to perform the functional assay [36,77].

Platelets from different donors vary considerably in their reactivity to HIT antibodies [19]. A potential explanation is the genetic polymorphism of the FcγRIIa receptor [78]. Two polymorphisms have been identified: Arg/His131 [79,80,81,82,83,84] and Gln/Lys127 [85]. It is of utmost importance to select platelets from responsive donors as it was shown that using a high responder donor for HIT investigation improves the sensitivity of the functional assay [86,87]. Indeed, platelets with poor reactivity may give false negative results with weak HIT antibodies [44,88]. Selection of platelets from a high responder donor seems to be the most important factor affecting the sensitivity of the HIT antibody functional assay [19]. In order to select responsive donors, different approaches have been adopted. Some laboratories use platelets from four different donors to minimize the variability of platelets. Some laboratories identify a number of “good responders” among the laboratory staff [19]. These known reactive donors are potential platelet donors when a HIT functional assay is needed. The identification can be realized in two ways. First, platelet donors may be tested individually against strong and weak positive control sera/plasma using a functional assay [27]. Platelet donors inducing a high platelet response with strong and with the weak positive control will be selected. Weak positive control sera/plasma are obtained from dilution of a well-characterized strong HIT sera/plasma [22]. The anti-CD9 monoclonal antibody ALB6 cross-links the FcγRIIa receptor and can be used as a platelet activator to select good donors [41,89,90]. The 5B9 is a chimeric IgG1 antibody to PF4/H complexes that mimics human HIT antibodies [91] which can potentially be used to select responsive donors.

Platelets should be obtained from donors that did not take medications impairing platelet function such as acetylsalicylic acid or non-steroid anti-inflammatory drugs (NSAIDs) [19,32]. Garlic, antihistamines, and naturopathic medications should also be avoided because of their effect on platelets [19,32,41].

For the use of HIMEA- and LTA-evaluating donor platelets aggregation, Morel-Kopp et al suggest to collect blood following ISTH-SSC recommendations [92]. These SSC/ISTH guidelines have been established for the standardization of LTA and recommend that treatment with drugs known to reversibly (e.g., NSAIDs except aspirin) or irreversibly (e.g., aspirin, thienopyridines) inhibit platelet function should be stopped at least 3 days and 10 days, respectively, before sampling [92]. Although, it is recognized in the literature that antiplatelet compounds should be avoided in donor blood, this is not systematically mentioned and minimum time between the last drug intake and the blood drawn may differ among papers [32,40,41,49,93]. Sono-Koree et al. evaluated the effects of aspirin and ibuprofen on donor platelets in responsive platelet donors to HIT antibodies using HPLC-SRA [27]. They concluded that the effects of these NSAIDs were donor specific and markedly decreased responses to HIT antibodies in several donors. They observed a return to normal platelet function in all donors by 1 week following drug ingestion and implemented a conservative approach, restricting SRA platelet donation in subjects who have ingested NSAIDs in the past 10–14 days [27].

Healthy donors with no platelet dysfunction should be selected to prevent false negatives. To ensure the good reactivity of platelets, donor platelets may be tested with common platelet activators such as collagen, ADP, arachidonic acid or thrombin receptor activating peptide (TRAP) during a pre-screening test [33,40]. Moreover, working with healthy donor blood decreases the risk of potential interfering substances that may cause false positives.

The number of donor(s) to perform a functional assay for the diagnosis of one suspected HIT patient varies from one to four among studies [18,27,41]. In general, studies using a high number of donors to minimize the variability of platelets selected them randomly [15,18]. This is less recommended as platelet donors are not easily available, therefore it may not be possible to obtain platelets from four donors to perform the assay on a regular basis [19]. Previous identification of highly responsive donors among the laboratory staff allows only one or two donors to be tested when a HIT functional assay is needed [18,19,41]. Some studies that worked with two or three donors known to be reactive to HIT antibodies did not test them separately but mixed their platelets to perform the HIT functional assay [15,27,58,94,95].

When performing the HIT assay with donor whole blood, blood group O or the same group as the patient should be used to avoid an ABO incompatibility reaction [40,56]. When working with PRP or washed platelets, ABO blood group discrepancies are inconsequential and may be ignored [22,31,36].

No international standardized guidelines for the appropriate selection of platelet donors to perform HIT functional assays currently exist. This may lead to interlaboratory variability [96].

## 5. Patient Sample

Functional assays may be performed with either patient serum or plasma [22,23,41]. An exception is the TGA requiring preferably plasma because it contains coagulation proteins that are needed for thrombin generation [61]. Testing should be performed using acute serum or plasma because HIT antibodies are transient [32]. Residual thrombin may contaminate patient serum or plasma and cause platelet activation leading to false positive results (e.g., patient with disseminated intravascular coagulation) [19,36]. The patient sample could be first heated at 56 °C for 30–45 min to inactivate thrombin [22,27]. Then, a high-speed centrifugation is performed (8000–12,000 g for 5–10 min) to remove fibrin(ogen)gel and other precipitates [23,32,36]. Complement proteins are also destroyed by heat inactivation but they are not required for IgG-dependent platelet activation in HIT [32]. However, overheating the patient sample can generate aggregated IgG causing platelet activation [19,23,36]. This heat-inactivation is not systematically performed for HIT functional assays in the literature. Morel-Kopp et al. do not recommend the heat inactivation of the patient sample before HIMEA testing because it may decrease antibody titers and affect platelet aggregation results [41].

## 6. Heparin

In vivo, unfractionated heparin (UFH) caused the HIT syndrome more frequently than low molecular weight heparin (LMWH) [16]. In vitro, studies demonstrated greater capacity of UFH to form highly immunogenic ultra large PF4/heparin/IgG complexes than LMWH [97]. In functional assays, UFH is commonly used [26,28,40,41,42], LMWH may be tested in parallel [18,25,58,98]. LMWH exhibits nearly 100% in vitro cross-reactivity to HIT antibodies using functional assays [27,48,99,100]. In others studies, LMWH showed less cross-reactivity compared with UFH, varying from low [101] to very high [102,103,104] cross-reactivity. These inconsistencies may be explained by a lack of standardization of test conditions such as source of donor platelets or optimal tested concentrations [102]. Two studies demonstrated that using reviparin, a LMWH, enhanced the sensitivity of the functional assay in comparison to UFH, because of the formation of more stable PF4/heparin complexes due to the narrow range in the molecular weight [98,105]. In the literature, reviparin or enoxaparin are used as LMWH in functional assays [18].

The concentration of heparin in the assay is critically important to induce maximal platelet activation/aggregation, maximizing the sensitivity of the assay [19]. Indeed, heparin and PF4 form heparin–antibody–PF4 complexes that activate platelets only over a narrow molar range [106,107]. Variable heparin concentrations are used in platelet activation assays for HIT diagnosis [18,88]. Pharmacologic heparin concentrations of 0.1 to 0.3 IU/mL are optimal for the formation of the heparin/PF4 complex in washed platelet-based assays [23]. For PRP-based assays, the optimal heparin concentrations are between 0.5 and 1.0 IU/mL [19,102]. Whole blood assays commonly use 1.0 IU/mL as low heparin concentration [39,40,41,42,43,56]. The rationale for using higher concentrations of heparin in whole blood and PRP-based assays compared to washed platelet-based assays is the presence of heparin binding proteins in plasma [88]. Multiple low heparin concentrations comprised in the optimal range may be tested in parallel during the same functional assay [18,22]. A high heparin concentration condition is recommended to enhance the specificity of the assay [19]. Indeed, high dose heparin disrupts the PF4/heparin complex [108] by saturating the heparin binding sites of all PF4 molecules. It suppresses platelet activation induced by HIT antibodies [19]. The high heparin concentration commonly used for washed platelet-based assays is 100 IU/mL [18,22,27]. In PRP-based assays and in whole blood-based assays, heparin at 100 IU/mL may not always completely suppress the HIT antibody-induced platelet activation because of the presence of heparin-binding proteins [19]. This partial inhibition may be acceptable or a higher concentration of heparin (i.e., 200 to 500 IU/mL) may be used to obtain a higher inhibition of the HIT-mediated platelet reaction [19,41,56].

Contaminating heparin in the patient sample can interfere with assay performances by inducing inappropriate final heparin concentrations in the assay [109,110]. Heparinase [110,111], resin for anions [58,112] or thiophilic adsorption chromatography [113] have been used to remove heparin contamination in the patient sample. Elimination of residual heparin in patient samples before performing a HIT functional assay is not a common practice in HIT diagnosis. In order to prevent an erroneous result caused by contaminating heparin, Morel-Kopp et al. recommend, whenever possible, to collect blood at least 4 h after cessation of an unfractionated heparin infusion and at least 12 h after a dose of LMWH [41].

## 7. Controls

Functional assays require strict quality controls [68]. Several controls have been described in the literature but they are not systematically performed [88]. There is a high variability of controls tested between laboratories.

### 7.1. Negative Controls

An appropriate negative control is needed to ensure the absence of platelet activation/aggregation of donor platelets in the presence of heparin with a negative sample [68]. To perform this negative control, the test sample may be replaced by a healthy control sample, a previously tested negative patient sample or a commercially available negative control sample [88].

The buffer control is a test condition performed in the absence of added heparin to prove the absence of donor platelets response with the patient sample without heparin [114].

### 7.2. Heparin Dependency

To verify that platelet activation/aggregation is FcγRIIa receptor-dependent and to prove the heparin dependency, two test conditions may be performed as a confirmation step [18]. First, the high heparin concentration condition disrupts PF4/heparin complexes and prevents the antibody-induced platelet activation response [18]. Second, the monoclonal antibody IV.3 blocks the FcγRIIa receptor and inhibits HIT-antibody-mediated platelet activation [18,19]. Heparin at high concentration or monoclonal antibody IV.3 should be added before donor platelets [18]. IV.3 is tested in the presence of a low heparin concentration [22], proving that the heparin dependency enhances the specificity of the assay [19,36].

### 7.3. Positive Controls

An appropriate positive control is required to verify that the platelets are sufficiently reactive and to ensure that the assay can adequately detect platelet-activating antibodies [68,88,115]. A known HIT-positive serum/plasma sample should be tested in parallel as standard control response [19]. HIT-positive serum/plasma of well-documented HIT are stored frozen and remain stable for many years [68]. Studies revealed that HIT sera stored at −70 °C continue to react well when used in the SRA or enzyme-immunoassay (EIA) more than two decades later [32,116,117]. The use of one or more “strong positive” and one or more “weak positive” HIT sera/plasma have been recommended [22,32,88,118]. “Strong positive” HIT controls are diluted to obtain “weak positive” HIT sera/plasma [22]. Weak-positive controls ensure the assay is sensitive enough to detect patients with HIT and assess variability in assay performance over time [22,118].

In theory, a HIT-mimicking monoclonal antibody may be used as a positive control [32]. For example, KKO, an IgG2bκ antibody [119] or 5B9, an IgG1 antibody (5B9) [91] are two monoclonal antibodies that activate human platelets through a heparin- and PF4-dependent mechanism that is mediated through FcγRIIA [91,119]. However, the correlation between the human platelets responsiveness and the reactivity to HIT antibodies needs to be evaluated to determine if a HIT-mimicking monoclonal antibody could be valuable in HIT diagnosis.

Platelet activating agonist controls may be used to ensure the good platelet responsiveness [88]. Heat-aggregated human IgG is sometimes used as an immune complex control added with and without IV.3 to ensure that the platelets respond to an “IgG agonist” and also to validate the FcγRIIa inhibition step as IV.3 inhibits platelet activation by heat-aggregated IgG [19,22,32,58]. Heat-aggregated IgG may be prepared from pooled normal sera by heat treatment at 63 °C for 20 min, followed by a 10-min centrifugation at 12,000 *g* [120]. Donor platelet reactivity may be tested in platelet aggregation assay with common platelet activators such as ADP, collagen, arachidonic acid or TRAP [33,40,41,77].

Some laboratories proposed a positive IgG-specific anti-PF4/heparin EIA as a quality control to avoid a false-positive SRA report as incongruous results may occur (i.e., positive SRA in combination with negative EIA and an atypical clinical presentation) [12,14,22,94,121,122].

## 8. Other Variations

Donor platelets are incubated with patient serum/plasma and heparin in all functional assays. This preanalytical step may differ for the agitation force, the incubation time and the incubation temperature among different functional assays or for the same functional assay. The incubation temperature may vary from room temperature [24,27,34,45] to 25–28 °C [48,51] to 37 °C [45,55]. To agitate, occasional gentle mixing [24,48], low speed [27], agitation of 1000 rpm [29,34,42] or 1200 rpm [55] are used. The incubation period may vary from 15 min [41,42] to 20 min [55,56] to 30 min [45,48,51] to 45 min [34] for up to 60 min [22,27,45]. The ratio of donor platelets to the patient sample is usually 3.75:1 for washed platelet-based assays [22,27] and usually between 1:0.5 and 1:1 for PRP and whole blood-based assays [36,39,40,55]. To collect donor platelets, ACD, citrate or hirudin tubes are often used. ACD tubes are used for washed platelet-based assays [22,27,58]. Citrate tubes are commonly used for PRP or whole blood-based assays [19,39,42,44,55]. Hirudin tubes are often used preferably for HIMEA as it improves assay sensitivity [39,89] by avoiding issues of calcium concentrations affecting platelet response (and also the problem of calcium depletion in under-filled citrate tubes [41]). Heparin tubes are avoided in order to prevent increase of final heparin concentration in the test.

## 9. Results Expression

Results expression is specific to the endpoint and the technology used. Functional assays using the release of radiolabeled or unradiolabeled serotonin as an endpoint (^14^C-SRA, EIA-SRA, HPLC-SRA) often express the results as a percentage of serotonin release to account for inter- and intra-individual variability in platelet serotonin content [25,27]. Expression of raw serotonin values may be used [24,25,28]. Platelet activation assays measuring ATP release reported luminescence results in moles of ATP per amount of platelets [29]. HIPA results are expressed as the presence or absence of a visual platelet aggregation [34]. PAT results are expressed as the area under the aggregation curve [44] or as percentage of aggregation [40,123]. HIMEA results are most commonly expressed as the area under the aggregation curve [39,40,42,43]. The aggregation velocity and the lag-time may be also used [41,89]. Flow cytometry experiments measuring the expression of platelet activation markers express results as a percentage of activated platelets [46,48,49,50,124]. Flow cytometry assays that detect generated PMPs may express results as the amount or the percentage of PMPs [51,58]. For the FcγRIIa proteolysis assay, results are determined as the percentage of proteolysis [64]. Some studies express the final results as a ratio between results at the low heparin concentration and at the high heparin concentration [51,55,56,95] or as a ratio between results at the low heparin concentration and in the absence of heparin [61]. The ratio low/high heparin concentration takes into account the heparin dependency confirmation step in the final result expression but is not applicable to each functional assay as the result at the high heparin concentration may be zero in HIMEA for instance [41].

## 10. Results Interpretation

According to the results of the test condition and controls, several situations are possible (Table 1).

In the first situation, the results of the functional assay are negative in the absence of added heparin (buffer control), at the low heparin concentration(s), at the high heparin concentration and with the monoclonal antibody IV.3. This is observed in the case of a true negative, i.e., the patient has really no HIT or in the case of a false negative. The latter may occur if the donor platelets were not sufficiently reactive because the donor is not a good responder to HIT antibodies or because he/she took antiplatelet agents. Another potential cause is a problem that occurred during the experiment. To avoid a false negative, it is highly important to obtain platelets from one or more good platelet responder that has been selected previously. Positive controls performed in parallel are essential to verify that donor platelets are optimally reactive and that the donor did not fail to mention antiplatelet compounds intake in the previous days [22]. Positive controls ensure that no technical problem occurs during the experiment and that the assay can adequately detect platelet activation or aggregation [22,68,88,115].

The second situation occurs in the case of a true HIT because of a positive reaction that occurs only with the low heparin concentration(s). Absence of platelet reaction with the high heparin concentration and the monoclonal antibody IV.3 conditions prove that the platelet reaction is heparin-dependent. The negative response in the absence of added heparin demonstrates that heparin is needed to activate/aggregate platelets.

In the third situation, the difference with the HIT laboratory profile is a positive response occurring in the buffer control. There are at least two potential explanations for strong platelet activation/aggregation in the absence of added heparin along with an inhibition at high heparin concentration and by FcγRIIa blocking antibody [36,113,125,126,127,128,129,130,131]. First, residual heparin may be present in the patient sample [109,110]. Second, HIT antibodies that activate platelets even in the absence of heparin may exist in the patient sample [96,113]. These antibodies are generated in four syndromes of autoimmune HIT: delayed-onset HIT, persisting HIT, spontaneous HIT syndrome and fondaparinux-associated HIT [125]. Platelet activation occurs in the absence of heparin because the HIT antibodies recognize PF4 bound to platelet-associated chondroitin sulfate [125,132]. Delayed-onset HIT indicates HIT that begins or worsens despite stopping heparin [125,133,134,135]. Persisting HIT is a HIT syndrome that takes several weeks to recover [125,128]. Spontaneous HIT syndrome is a disorder clinically and serologically resembling HIT but without proximate heparin exposure [125,126,127,136,137]. Fondaparinux-associated HIT is a HIT syndrome that occurs during fondaparinux treatment, the causal association of fondaparinux and HIT is controversial [138]. These disorders highlight the need of the buffer control to diagnose them from a typical HIT [114].

In the fourth situation, we observe a platelet activation/aggregation with each condition except at the high heparin concentration. The platelet response is not inhibited by monoclonal antibody IV.3 but only by a high heparin concentration. This may be mediated by residual thrombin [58,94]. Indeed, thrombin is a potent platelet activator [64]. This activation progressively decreases from buffer control to low heparin concentration to high heparin concentration [36]. To prevent this false positive, heat-inactivation of the patient sample at 56 °C before performing the assay is performed [22,27]. Another laboratory practice to inactivate thrombin is to add hirudin to the patient sample [18,34,36,94]. These results may also be obtained with strongly-reacting HIT sera/plasma [94]. Some very strong HIT samples may induce platelet activation/aggregation that is not inhibited by monoclonal antibody IV.3 but with a high heparin concentration [36,94]. These strongly-reacting HIT antibodies are able to activate platelets without heparin [122].

In the fifth situation, a platelet response is observed in each condition except with the monoclonal antibody IV.3. A potential explanation is the presence of immune complexes in the patient sample such as heat-aggregated human IgG [22,77,98]. Heat-aggregated IgG may be produced during an inappropriate heat-inactivation of the patient sample [19,23,36]. They induce FcγRIIa-dependent platelet activation even in the presence of high heparin concentration but which is inhibited by the monoclonal antibody IV.3 [98,139]. The reaction profile may also occur in the presence of high-titer HLA class I alloantibodies as they react with HLA class I antigens present in high density on platelets [36]. Systemic lupus erythematosus or other platelet-activating factors may also be responsible for this situation [36,78,140]. Inhibition by IV.3 is not entirely specific for the HIT antibody-induced platelet activation [19].

The sixth reaction pattern presents a positive platelet reaction at each condition. This may be caused by very strong HIT antibodies that activate platelets in the presence of monoclonal antibody IV.3 but even with a high concentration of heparin [36,68,141].

Thrombotic thrombocytopenic purpura may lead to variable activation in the presence of heparin that is not inhibited by FcγRIIa-blocking monoclonal antibody [32,63,78]. It has been mentioned that elevated acute-phase reactant proteins such as fibrinogen, commonly present in plasma from critically ill patients, may cause heparin-dependent platelet aggregation in PAT [69,142,143].

The result of a functional assay is considered positive if a positive reaction occurs with the low heparin concentration(s) and is inhibited by the high heparin concentration and by monoclonal antibody IV.3 (pattern 2 and 3) [19]. The result is considered negative if no platelet activation occurs with no, low and high heparin concentration and with monoclonal antibody IV.3 (pattern 1) along with an appropriate reaction of the positive controls. When samples cannot be classified as either positive or negative, they are designated as “indeterminate results” (patterns 4, 5 and 6) [22]. In these indeterminate reactions, platelet activation/aggregation occurs with low heparin concentration but is not inhibited by high heparin concentration and/or IV.3 [64]. Facing an indeterminate result, strategies have been proposed in the literature. The first solution is to repeat the experiment with another properly heat-inactivated sample [19]. On the one hand, a sample not sufficiently heated may still be contaminated by residual thrombin and induce pattern 4. On the other hand, an overheated sample may contain heat-aggregated IgG and be responsible of pattern 5 [27]. An interpretable result may be obtained when the assay is repeated using another heat-inactivated aliquot [94]. The second solution (that may be coupled with the first solution) is to repeat the assay using different platelet donors; the subsequent test may yield a clear negative or positive result [32,94]. An explanation is that platelet activation by immune antibodies such as HLA or thrombotic thrombocytopenic purpura antibodies may be dependent on donor platelet phenotype [27]. The third solution is to take into account the result of an anti-PF4/heparin EIA to assess for the presence or absence of anti-PF4/heparin antibodies in indeterminate samples [12,36,94]. EIA assays have a high sensitivity and a negative result essentially rules out HIT [68]. They are often performed before functional assays as recommended in diagnosis algorithms [5,14]. In case of a positive EIA, the fourth solution is to retest dilutions of patient samples. A clear HIT pattern of reactivity may be obtained and indicates that very strong HIT antibodies are present [22,94].

The possible different reaction pattern, caused by other factors than HIT antibodies able to activate platelets, highlights the importance of running appropriate controls when performing a functional assay for the diagnosis of HIT.

## 11. Threshold and Performances

Different ways to determine a threshold in the literature are used to separate positive and negative platelet reactions. A cut-off may be defined in the laboratory because of its traditional use in the literature [22,27,144,145]. This is often the case with SRA whose threshold of 20% was historically defined by Sheridan et al. [23]. However, some authors favor the use of a higher cut-off of 50% [3,22,32,146] as this better discriminates between HIT and non-HIT thrombocytopenia [3,36]. The mean negative control value + 2 SD [47,112,147] or + 3 SD [24,42] may be used as the threshold. Some papers suggest that each laboratory should determine its own cut-off based on negative control using local donors [41,56]. A receiver operating characteristic (ROC) curve analysis is sometimes performed to determine the threshold value that gives maximal sensitivity and specificity [24,40,95]. Because functional tests are not used as screening tests but as confirmatory tests, a better specificity should be preferred over a better sensitivity [26,42]. The ROC curve of a functional assay is obtained against a reference standard. Functional assays, SRA [24,26,42,44,48] or HIPA [95] are sometimes used as reference in the literature to calculate the performances of the studied functional assay. More recently, the diagnosis of HIT based on the opinion of two or three independent experts was used as the reference standard [40,43,56,115,121,124,148]. Experts use all available clinical information, including follow-up data on each patient with HIT suspicion to make the diagnosis blinded to the results of the laboratory tests [115]. This expert consensus diagnosis has been questioned in the literature [115,149]. The combination of SRA with EIA has been proposed as a reference standard [68,149]. Clinico-biological conclusion combining a biological result, such as SRA or HIPA, with clinical parameters has also been used or proposed as a reference standard [65,115,150]. No universally accepted reference standards to measure performances of functional assay currently exist [7,151]. Since the studies do not use the same reference standard, clear-cut definitions of the specificities and sensitivities of the available functional assays are not given [69]. Moreover, even for the same functional assay, interlaboratory differences in methodology exist and performances reported by one laboratory do not necessarily apply to others [22].

## 12. Existing Functional Assays and Their Characteristics

SRA and HIPA are two washed platelet-based assays, often considered as reference standards for diagnosing HIT [14,18] although no universally accepted gold standard for HIT exists [65]. The reported sensitivity and specificity of SRA and HIPA are over 95% [115,151,152]. HIPA requires no special equipment and a moderate level of expertise but its activation endpoint is evaluated subjectively with possible visual interferences (Table 2) [14,36,69].

SRA is not available in most routine hospital laboratories because it requires the use of radioactive material with expensive special equipment and a specific license [36,38]. This assay needs a high level of expertise and is time-consuming [22]. SRA is available to most clinicians only as send-outs to highly specialized laboratories and it does not provide results in real time necessary to guide initial management [14,148,152]. Moreover, even if SRA is available in the laboratory, the result may be obtained with a delay of several days [2]. SRA is not applicable for immediate patient management but rather for an ultimate HIT diagnosis [22]. Moreover, it has been reported that 10% of samples tested for HIT with SRA are initially classified as indeterminate which further delays accurate diagnosis [64,94]. SRA may also be performed with PRP [65,153] but this is a less common practice [22,112]. Because most of the laboratories try to avoid radioactivity for regulatory and safety issues [1] and because a rapid assay is very desirable in HIT diagnosis, researchers developed and evaluated new techniques. Alternative “non-radioactive serotonin-release assays” have been proposed in the literature using ELISA [24,25,26], HPLC [25,26,27] or flow cytometry [28]. A common advantage of these techniques is that they measure endogenous serotonin, avoiding the ^14^C-serotonin platelet preloading step of SRA which simplifies the preanalytical procedure. ELISA does not require special equipment except a microplate spectrophotometer but it is a time-consuming assay procedure [24,25]. HPLC and flow cytometry are special equipment, with a high initial capital cost, that require technical expertise but these technologies offer a larger availability compared to a radioactive assay [26]. Studies that compared non-radioactive serotonin-release assay to SRA demonstrated similar performances [24,27] but further evaluation is needed. PAT was the first HIT assay described in the literature [37]. PAT has the advantage of easy handling but its sensitivity and specificity were demonstrated to be inferior to SRA/HIPA even when good platelet donors were selected [1,14,33,42,69]. HIMEA performed with whole blood proved to be a more sensitive and specific assay than PAT [40,42,89] and showed similar performances to SRA [40,42,44,89]. This semi-automated assay has the advantage of being easy-to-perform, requiring a moderate level of expertise. The equipment needed is a multiple electrode platelet aggregometer, widely used for antiplatelet treatment monitoring [154] and largely available in laboratories [39]. Its rapid turnaround time [41,42] and large availability reduce the time taken to confirm a HIT diagnosis and should have a positive impact on patient management [41]. Working with whole blood does not require platelet handling and preparation but has the limitation of needing a compatible blood group donor to avoid an ABO response [40]. More studies are needed to confirm the equivalence of HIMEA to SRA/HIPA [41]. A standard HIMEA protocol has been proposed by the SSC of the ISTH to serve as a standard for multicenter studies [41]. ATP release assay is a rapid and easy-to-perform assay that has been evaluated in one paper for HIT diagnosis [29]. Concordance with SRA was very good but needs further evaluation [29]. Flow cytometry assays (FCA) measuring platelet activation markers have been proposed in HIT diagnosis [45,46,47,48,49,50,51,95,124]. Studies showed good correlation between FCA and SRA [48,49], HIPA [50,95] or final clinical HIT diagnosis [124]. Flow cytometry measuring PMPs was evaluated as a functional assay [51,55,56,57,58]. Washed platelets [58], whole blood [55,56,57] or PRP [51] were used. Studies showed that PMPs as a platelet activation endpoint gave comparable results with SRA [55,56,58]. Further studies evaluating flow cytometry in the diagnosis of HIT are needed [14]. FCA requires high technical expertise and a high initial outlay on expensive equipment; however, this is cost-effective [48,51,95] and rapid [48,50,51,95,124]. TGA was investigated in the research setting in one study [61]. Results of the TGA correlated well with the results of PAT. They concluded that generation of thrombin could potentially be used for the diagnosis of HIT but needs further evaluation [14]. FcγRIIa proteolysis was shown to be at least as specific as the SRA for the diagnosis of HIT [64]. This endpoint has the advantage of being specific for FcγRIIa-mediated platelet activation [64]. For example, thrombin is a potent platelet activator that will not cause proteolysis of FcγRIIa [64]. DT40-luciferase was proposed as a functional cell-based assay not requiring donor platelets [65]. The cell line may be stored at −80 °C and retrieved as needed. This assay showed better discrimination than two commercial immunoassays. It is easy-to-perform but not widely available. Stability of the transfected cell line and larger prospective validation are needed.

Specialized laboratories use mainly ^14^C-SRA, HIPA, PAT or HIMEA as a functional assay to diagnose HIT. Other methods presented in Table 2 are more used in a research perspective but they may become more available for routine use. Indeed, the evolution of technology and the reduction of the equipment cost can render some sophisticated techniques more accessible, such as HPLC (HPLC-SRA) or flow cytometry (FCA-membrane GP, PMPGA, FCA-intraplatelet serotonin).

## 13. Conclusions

An ideal functional assay would be easy-to-perform, rapid, widely available in real-time, standardized and would have excellent performance. On-demand HIT testing has the potential to have a positive clinical and economic impact [155]. In confirmed HIT patients, it improves clinical outcomes by enabling earlier appropriate treatment and reduce costs by preventing expensive complications. In non-HIT patients, it could reduce overdiagnosis, unnecessary treatment and replacement anticoagulant drug costs [65,155]. Practically, few laboratories are currently able to perform a functional assay [2,42]. Among laboratories performing functional assays, there is currently a high variability in pre-analytical sample preparation and handling [22], platelet donors selection, controls performed, heparin concentrations used, testing methodologies and results interpretation [88]. The variability in HIT functional assays among laboratories reflects the lack of consensus recommendations on HIT testing and indicates a need for proficiency testing to assess assay performances [88]. Functional assays with few technical limitations facilitate their standardization and increase their accessibility in laboratories. Further standardization and evaluation of functional assays based on consensus guidelines would be valuable for a rapid and accurate diagnosis of HIT.

## Figures and Tables

**Table 1:** Different patterns with a combination of functional assay results (platelet response) at four test conditions (i.e., absence of added heparin, low concentration(s) and high concentration of heparin and monoclonal antibody IV.3). Potential causes of each pattern are provided. Negative and positive controls are not represented in the table. Neg: negative, Pos: positive, Ab: antibody, IgG: immunoglobulin G, HLA: human leukocyte antigen.

Pattern	Absence of Added Heparin	Low Concentration(s) of Heparin	High Concentration of Heparin	Monoclonal Ab IV.3	Potential Causes
**1**	Neg	Neg	Neg	Neg	No HIT HIT and low platelet reactivity Technical problem
**2**	Neg	Pos	Neg	Neg	HIT
**3**	Pos	Pos	Neg	Neg	HIT with residual heparin Syndromes of autoimmune HIT: Delayed-onset HIT Persisting HIT Spontaneous HIT syndrome Fondaparinux-associated HIT
**4**	Pos	Pos	Neg	Pos	Residual thrombin Very strong HIT
**5**	Pos	Pos	Pos	Neg	Heat-aggregated IgG High-titer HLA class I alloantibodies Systemic lupus erythematosus Other platelet-activating factor
**6**	Pos	Pos	Pos	Pos	Very strong HIT

**Table 2:** Functional assays described in the literature for the diagnosis of HIT and associated technique/technology, studied endpoint, platelet suspension, advantages and limitations. SRA: serotonin-release assay, ^14^C: carbon-14, EIA: enzyme-immunoassay, HPLC: high-pressure liquid chromatography, FCA: flow cytometry assay, HIPA: heparin-induced platelet activation, PAT: platelet aggregation assay, HIMEA: heparin-induced multiple electrode aggregometry, ATP: adenosine triphosphate, PMPGA: platelet microparticle generation assay, TGA: thrombin generation assay, PRP: platelet rich plasma, GP: glycoproteins, PMPs: platelet microparticles.

Assay	Technique/Technology	Endpoint	Platelets Used	Advantages	Limitations
**^14^C-SRA**	β-counter	^14^C-radiolabeled serotonin release from dense granules of activated platelets	Washed platelets (PRP)	High sensitivity High specificity	Time-consuming High technical expertise Radioactivity and specific license Expensive equipment Limited availability
**EIA-SRA**	ELISA	Serotonin release from dense granules of activated platelets	Washed platelets	Endogenous serotonin No radioactive serotonin preloading No special equipment needed Quantitative determination of serotonin	Time-consuming
**HPLC-SRA**	HPLC	Serotonin release from dense granules of activated platelets	Washed platelets	Endogenous serotonin No radioactive serotonin preloading Rapid Quantitative determination of serotonin	High technical expertise Expensive equipment Not widely available
**FCA-intraplatelet serotonin**	Flow cytometer	Loss of intraplatelet content of serotonin from activated platelets	PRP	Rapid Reproducible	High technical expertise Expensive equipment Not widely available
**HIPA**	Visual observation	Visual assessment of platelet aggregation	Washed platelets	High sensitivity High specificity No special equipment needed Repeated evaluation of platelet activation over time Moderate level of expertise required Moderate time consumption	Subjective visual assessment Possible interference with visual interpretation
**PAT**	Aggregometer	Change of light transmittance caused by platelet aggregation	PRP	Largely available equipment in laboratory Easy-to-perform Objective assessment of platelet aggregation Record over time	Low sensitivity Moderate specificity
**HIMEA**	Multiple electrode platelet aggregometry	Changes in impedance caused by platelet aggregation on electrodes	Whole blood	Easy-to-perform Semi-automated No platelet handling and preparation Moderate level of expertise required Rapid Largely available equipment in laboratory	Compatible blood group donor
**ATP release assay**	Lumiaggregometer/Standard scintillation counter	Detection of ATP release from activated platelets	Washed platelets PRP	Easy-to-perform Rapid	Not widely available
**FCA-membrane GP**	Flow cytometer	Expression of platelet activation markers (anionic phospholipids or P-selectin) in platelet population (CD61 or CD41)	PRP	Rapid Cost-effective	Expensive equipment High technical expertise Not widely available
**PMPGA**	Flow cytometer	Generation of PMPs	Washed platelets Whole blood PRP	Rapid Cost-effective	Expensive equipment High technical expertise Not widely available
**TGA**	Fluorometer	Generation of thrombin	PRP		
**FcγRIIa proteolysis assay**	Western blot/densitometer	Proteolysis of FcγRIIa	Washed platelets	Specific for FcγRIIa-mediated platelet activation	Not widely available
**DT40-luciferase**	Luminometer	Luciferase activity induced by cell activation	Platelet substitutes: chicken B lymphocytes	No need of donor platelets Cell line stored at −80 °C and retrieved as neededEasy-to-perform	Not widely available
